# Basic Review of the Cytochrome P450 System

**DOI:** 10.6004/jadpro.2013.4.4.7

**Published:** 2013-07-01

**Authors:** Anne M McDonnell, Cathyyen H. Dang

**Affiliations:** From Brigham and Women’s Hospital, Boston, Massachusetts, and Froedtert & The Medical College of Wisconsin, Milwaukee, Wisconsin

Cytochrome P450 (CYP) is a hemeprotein that plays a key role in the metabolism of drugs and other xenobiotics (Estabrook, 2003). Understanding the CYP system is essential for advanced practitioners (APs), as the consequences of drug-drug interactions can be profound. In this article, we will describe the CYP system, its potential for drug interactions, and the genetic polymorphisms that can exist in hematology/oncology patients.

Drug metabolism occurs in many sites in the body, including the liver, intestinal wall, lungs, kidneys, and plasma. As the primary site of drug metabolism, the liver functions to detoxify and facilitate excretion of xenobiotics (foreign drugs or chemicals) by enzymatically converting lipid-soluble compounds to more water-soluble compounds. Drug metabolism is achieved through phase I reactions, phase II reactions, or both. The most common phase I reaction is oxidation, which is catalyzed by the CYP system (Gibson & Skett, 2001).

Klingenberg first discovered CYP in 1954 during his research on steroid hormone metabolism, when he extracted a novel protein from hepatocytes (Klingenberg, 1958). It was almost a decade later that the function and significance of CYP were determined. In 1963, Estabrook, Cooper, and Rosenthal described the role of CYP as a catalyst in steroid hormone synthesis and drug metabolism. Cooper and colleagues later confirmed CYP to be a key enzyme involved in drug and steroid hydroxylation reactions (Cooper, Levin, Narasimhulu, Rosenthal, & Estabrook, 1965). Numerous CYP proteins have since been discovered and found to be widespread throughout the body, demonstrating significant involvement in chemical activation, deactivation, and carcinogenesis (Estabrook, 2003).

## Classification

Cytochrome P450 pathways are classified by similar gene sequences; they are assigned a family number (e.g., CYP1, CYP2) and a subfamily letter (e.g., CYP1A, CYP2D) and are then differentiated by a number for the isoform or individual enzyme (e.g., CYP1A1, CYP2D6). Drugs that share a common pathway have the potential for drug-drug interactions (Nelson, 2009). The classification of CYP proteins will be the APs first hint of the potential for drug interactions. Not all drugs have CYP activity. However, drugs with CYP activity may be inhibitors, inducers, or substrates for a specific CYP enzymatic pathway, thus altering the metabolism of concurrently administered agents. Drugs that inhibit an enzymatic pathway of CYP may cause increased concentrations of other drugs metabolized by the same pathway, resulting in drug toxicity. Likewise, drugs that induce an enzymatic pathway of CYP may reduce concentrations of drugs metabolized by the same pathway, leading to subtherapeutic drug levels or treatment failure.

A 2008 review of the most commonly sold prescription drugs in the United States reported that the majority of hepatically cleared drugs involved the CYP enzymes from families 1, 2, or 3 (Zanger, Turpeinen, & Schwab, 2008). The most common pathways involved, CYP3A4/5, CYP2C9, CYP2D6, and CYP2C19, accounted for approximately 79% of these drugs’ oxidation (Zanger, Turpeinen, & Schwab, 2008). Although only one chemotherapy agent was listed in the top 200 list, many patients have medical comorbidities that warrant concomitant drug therapies, which can then lead to drug-drug interactions. Therefore, it is important to understand the CYP system for both chemotherapy and nonchemotherapy agents.

## Drug-Drug Interactions

A consequence of drug-drug interactions may include the augmentation of known potential side effects. Imatinib (Gleevec) is an oral tyrosine kinase inhibitor that is approved by the US Food and Drug Administration (FDA) for the treatment of Philadelphia chromosome–positive acute lymphoblastic leukemia and chronic myelogenous leukemia (Novartis Pharmaceuticals, 2012). Because imatinib is both a CYP3A4 substrate and inhibitor, caution should be taken when CYP3A inhibitors and CYP3A inducers are concurrently prescribed. CYP3A inhibitors, such as azole antifungals, can increase imatinib concentrations; CYP3A inducers, such as rifampin, can decrease imatinib levels, leading to either supra- or subtherapeutic levels of imatinib, respectively (Haouala et al., 2011; Novartis Pharmaceuticals, 2012; FDA, 2011; The Medical Letter of Drugs and Therapeutics, 2011). The HMG-COA reductase inhibitor simvastatin is a CYP3A4 inhibitor with the potential for dose-related myopathies. Concurrent use of simvastatin with imatinib may increase imatinib levels in the body while also increasing simvastatin drug levels (FDA, 2011)

Not all CYP-mediated drug interactions are clinically significant. The clinical significance of CYP-mediated drug interactions can be more concerning among drugs with a narrow therapeutic window. This may require dosage adjustments for one or more agents. For example, temsirolimus (Torisel), which is an IV mTOR inhibitor, is approved in the treatment of advanced renal cell carcinoma and is metabolized by the CYP3A4 pathway (Pfizer, 2012a). The manufacturer recommends doubling the dose of temsirolimus when used concurrently with strong CYP3A4 inducers such as phenytoin or fosphenytoin (Pfizer, 2012a). It should be noted that the drugs may still interact, despite different routes of administration.

Another example of an agent with a narrow therapeutic window is tacrolimus, which is used at some centers for the non–FDA-approved indication of immunosuppressant in hematopoietic stem cell transplant. Concurrent use of tacrolimus and omeprazole, a substrate for both CYP2C19 and CYP3A4, can result in increased tacrolimus concentrations, resulting in supratherapeutic levels and increased tacrolimus toxicity (Astellas-Pharma US, 2012). Additionally, voriconazole, a triazole antifungal, is known to have a clinically significant drug interaction with tacrolimus. Due to voriconazole’s CYP3A4 inhibition, the dose of tacrolimus when given in combination with voriconazole is one-third of the usual recommended starting dose (Pfizer, 2012b). The inhibitory effects of voriconazole are not limited to CYP3A4. In vitro tests have also shown voriconazole to inhibit the CYP2B6, CYP2C9, and CYP2C19 enzyme pathways (Jeong, Nguyen, & Desta, 2009). This highlights the ability for a single drug to have activity in more than one CYP pathway.

As our understanding of the CYP system has improved, new agents have extensive drug interaction studies performed before they reach the market. However, not all agents have been tested in combination. Sometimes, drug interactions are hypothesized based on known metabolic pathways. For example, tamoxifen is an estrogen receptor antagonist approved for use in patients with breast cancer. Its metabolism is complex and involves a number of CYP pathways, starting with activation through metabolism. However, CYP2D6 appears to be the most significant in the production of the active metabolite endoxifen (Brauch, Murdter, Eichelbaum, & Schwab, 2009). It follows that CYP2D6 inhibitors may cause decreased production of endoxifen, resulting in treatment failures. It has been proposed that selective serotonin reuptake inhibitors (SSRIs) with potent CYP2D6 inhibitory activity may lead to decreased tamoxifen activity in patients with breast cancer.

An observational study evaluated patients on concurrent CYP2D6 inhibitors of varying potential. However, they did not find a difference in survival between patients who took a CYP2D6 inhibitor, regardless of strength, and those who did not (Dezentjé et al., 2010). This differs from the results of a retrospective cohort, which evaluated women on tamoxifen who were treated with an SSRI. Kelly et al. (2010) reported that women treated with paroxetine, a potent CYP2D6 inhibitor, were more likely to die from breast cancer, possibly due to treatment failure as a result of the tamoxifen-paroxetine drug interaction. Due to the conflicting data and the potential risks of treatment failure, the National Comprehensive Cancer Network (NCCN) treatment guidelines currently recommend clinicians use caution with concurrent strong CYP2D6 inhibitors and tamoxifen (NCCN, 2013).

Effects of CYP on drugs are not only limited to hepatic metabolism but also present in drug absorption by the small intestine. Drugs absorbed from the small intestine often undergo first-pass metabolism mediated by CYP3A4. Grapefruit juice, an inhibitor of CYP3A4, acts locally on the small intestine and inhibits enterocyte CYP3A4, which in turn results in higher systemic levels of CYP3A active drugs (Bailey & Dresser, 2004; Bailey, Malcolm, Arnold, & Spence, 1998; Kato, 2008) such as imatinib (Novartis Pharmaceuticals, 2012).

Substrates of common chemotherapeutic agents are listed in Table 1. Table 2 lists common hematology- and oncology-related CYP inhibitors and inducers. A complete classification of the CYP activity for all available drugs is beyond the scope of this publication. However, tertiary online references such as the Indiana School of Medicine’s P450 drug interaction table can be accessed at http://medicine.iupui.edu/clinpharm/ddis/table.aspx (Indiana University, 2012). Additionally, general drug interaction information can be found at www.drugs.com.

**Table 1:**
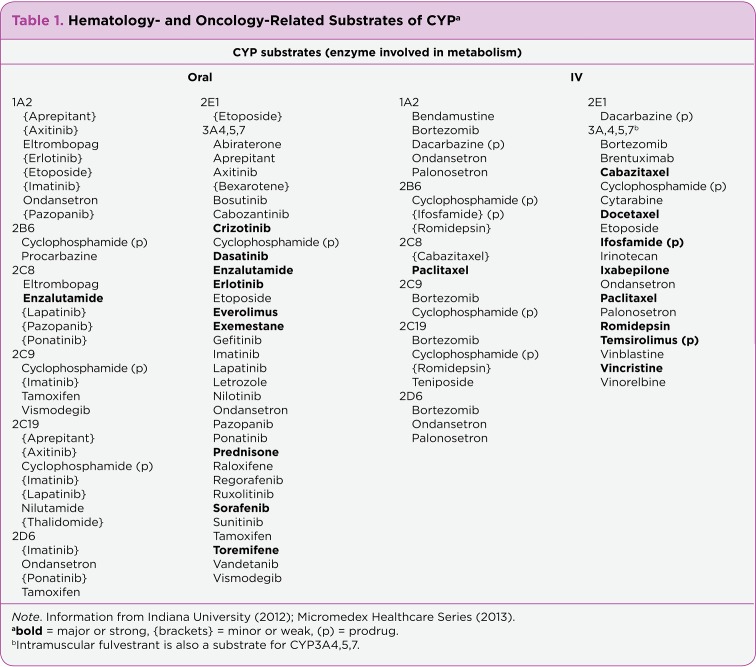
Table 1. Hematology- and Oncology-Related Substrates of CYP

**Table 2:**
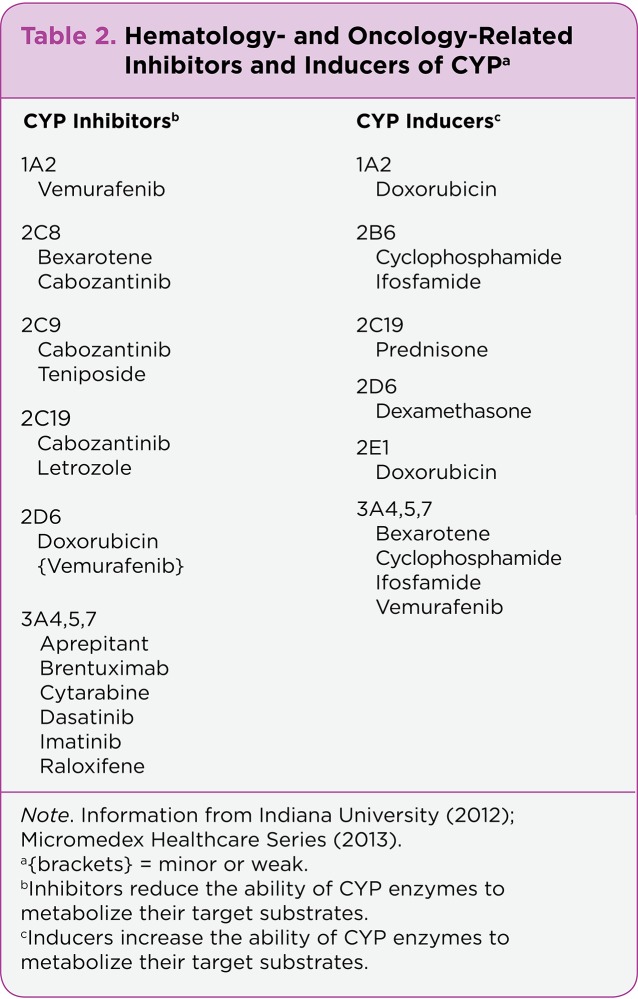
Table 2. Hematology- and Oncology-Related Inhibitors and Inducers of CYP

## Variation Among Patients

Drug interactions are not the only potential pitfalls related to CYP. Genetic mutations or polymorphisms (genetic variants) of CYP are known to exist among patients. Depending on the phenotype encoded by these genes, the metabolism of certain drugs may be variable. Each person’s ability to metabolize drugs is determined by the pairing of individual alleles he or she has inherited from his or her parents. Each allele may be categorized as a wild-type (functional) or variant (defective) allele. Wild-type alleles are considered "normal" and occur predominantly in the general population, whereas variant alleles may confer diminished or no activity. People who carry two wild-type alleles will generally have "normal" rates of metabolism (extensive metabolizers), whereas a person who carries two variant (defective) alleles will inherently have little to no enzyme activity (poor metabolizers). Those who inherited one of each allele will have decreased enzymatic activity (intermediate metabolizers). In certain cases, when gene duplication or amplification results in more than two gene copies of wild-type alleles, enzyme activity will be greater than normal (ultrarapid metabolizers; Johansson & Ingelman-Sundberg, 2011).

Genetic polymorphisms can have a significant impact on drug therapy and should be taken into consideration in clinical practice, especially when unexpected outcomes arise. For example, intermediate and poor metabolizers are at increased risk for toxicity and adverse effects due to drug accumulation. These patients demonstrate hypersensitivity or low tolerance to particular drugs and subsequently may require reduced doses or avoidance of the drug altogether. Acute dystonic reactions due to metoclopramide administration have been documented in patients with homozygous CYP2D6 variant alleles (van der Padt, van Schaik, & Sonneveld, 2006). Similarly, deaths have been reported in patients receiving methadone and fluoxetine, in separate cases. Autopsies revealed abnormally high drug concentrations that were attributed to the presence of CYP2B6 and CYP2D6 variant alleles, respectively (Bunten, Liang, Pounder, Seneviratne, & Osselton, 2010; Sallee, DeVane, & Ferrell, 2000). Conversely, prodrugs, defined as inactive parent drugs that require enzymatic conversion to the active metabolite, may exhibit low drug efficacy in poor metabolizers. These patients may need higher doses of drugs to produce the same response as extensive metabolizers.

Ultrarapid metabolizers represent the opposite end of the spectrum but may also be disposed to drug toxicity when the metabolite is more active than the parent drug. Codeine is metabolized to the active metabolite morphine via CYP2D6 and generally provides mild analgesic and cough suppressant effects. However, when codeine is administered to patients carrying CYP2D6 gene duplication, approximately 50% more morphine is produced (Kirchheiner et al., 2007). As a consequence of such amplification of drug effects, devastating outcomes have occurred. For example, a baby suffered from fatal morphine toxicity when her mother was prescribed codeine while breastfeeding. Genotyping later showed that the mother carried an extra copy of the wild-type CYP2D6 gene (Madadi et al., 2007). Likewise, tramadol, another commonly prescribed analgesic drug, is metabolized via CYP2D6 to a more active agent. Respiratory depression and increased adverse effects have been reported in ultrarapid metabolizers (Stamer, Stuber, Muders, & Mushoff, 2008). Alternatively, effects of certain drugs may be diminished or short-acting due to rapid metabolism and deactivation in these patients.

In the past several years, tamoxifen has been widely discussed and researched, not only for potential drug interactions but also for polymorphic variations (Schroth et al., 2009; Regan et al., 2012). As mentioned previously, endoxifen, an active metabolite of tamoxifen, relies on CYP2D6 for its formation (Desta, Ward, Soukhova, & Flockhart, 2004; Brauch et al., 2009). It has been proposed that the genetic polymorphisms of CYP2D6 among extensive, intermediate, and poor metabolizers may lead to variable levels of endoxifen, which may lead to treatment failures (Desta et al., 2004; Schroth et al., 2009; Regan et al., 2012). A retrospective analysis of breast cancer patients taking tamoxifen reported that patients who were poor metabolizers relapsed faster than those who were not poor metabolizers (Schroth et al., 2009; NCCN, 2013). But research by Regan et al. (2012), which sought to clarify the role of CYP2D6 polymorphisms in women with breast cancer taking tamoxifen, demonstrated that risk of tumor recurrence was not associated with a specific polymorphism. At this time, conflicting data do not allow us to predict response to tamoxifen therapy based on CYP2D6 polymorphisms.

## Conclusions

CYP is a complex and important component of drug metabolism. It is the root of many drug interactions due to inhibition, induction, and competition for common enzymatic pathways by different drugs. Genetic variability of CYP is also a significant source of unpredictable drug effects. Awareness and understanding of drugs involved in common CYP pathways will add to the AP’s knowledge base to foresee and prevent potential drug interactions and untoward effects.

## Acknowledgments

The authors would like to thank Nancy J. Lee, PharmD, BCPS, and Oanh H. Dang, PharmD, BCPS, for their review and editing of this article.
